# Supplementary material for: Potent NTD-Targeting Neutralizing Antibodies against SARS-CoV-2 Selected from a Synthetic Immune System
Source: Vaccines (Basel). 2023 Mar 31;11(4):771. doi: 10.3390/vaccines11040771 (PMC10143083; doi:10.3390/vaccines11040771)
Supplement: Supplementary file 1 [file vaccines-11-00771-s001.zip › vaccines-2241176-supplementary.pdf]

Supplementary Material

Potent NTD-targeting neutralizing antibodies against SARS-CoV-2 selected from a synthetic immune system

Wenping Li [1, 2, 3, 4], Fulian Wang [1, 2, 3, 4], Yu Li[1, 2, 3, 4], Lei Yan [1], Lili Liu [1], Wei Zhu [1], Peixiang Ma [5], Xiaojie Shi [1, *], Guang Yang [1, *]

[1] Shanghai Institute for Advanced Immunochemical Studies, ShanghaiTech University, Shanghai, China

[2] School of Life Science and Technology, ShanghaiTech University, Shanghai, China.

[3] CAS Center for Excellence in Molecular Cell Science, Shanghai Institute of Biochemistry and Cell Biology, Shanghai, China

[4] University of Chinese Academy of Sciences, Beijing, China

[5] Shanghai Key Laboratory of Orthopedic Implants, Department of Orthopedic Surgery, Shanghai Ninth People's Hospital, Shanghai Jiao Tong University School of Medicine, Shanghai, China

* Correspondence: yangguang@shanghaitech.edu.cn (G.Y); Tel.: +86-21-20685030; shixj@shanghaitech.edu.cn (X.S)

Contents

Figure S1 3
Figure S2 4
Figure S3 5
Figure S4 6
Figure S5 7
Figure S6 8
Figure S7 9
Figure S8 10
Table S1 11

Figure S1

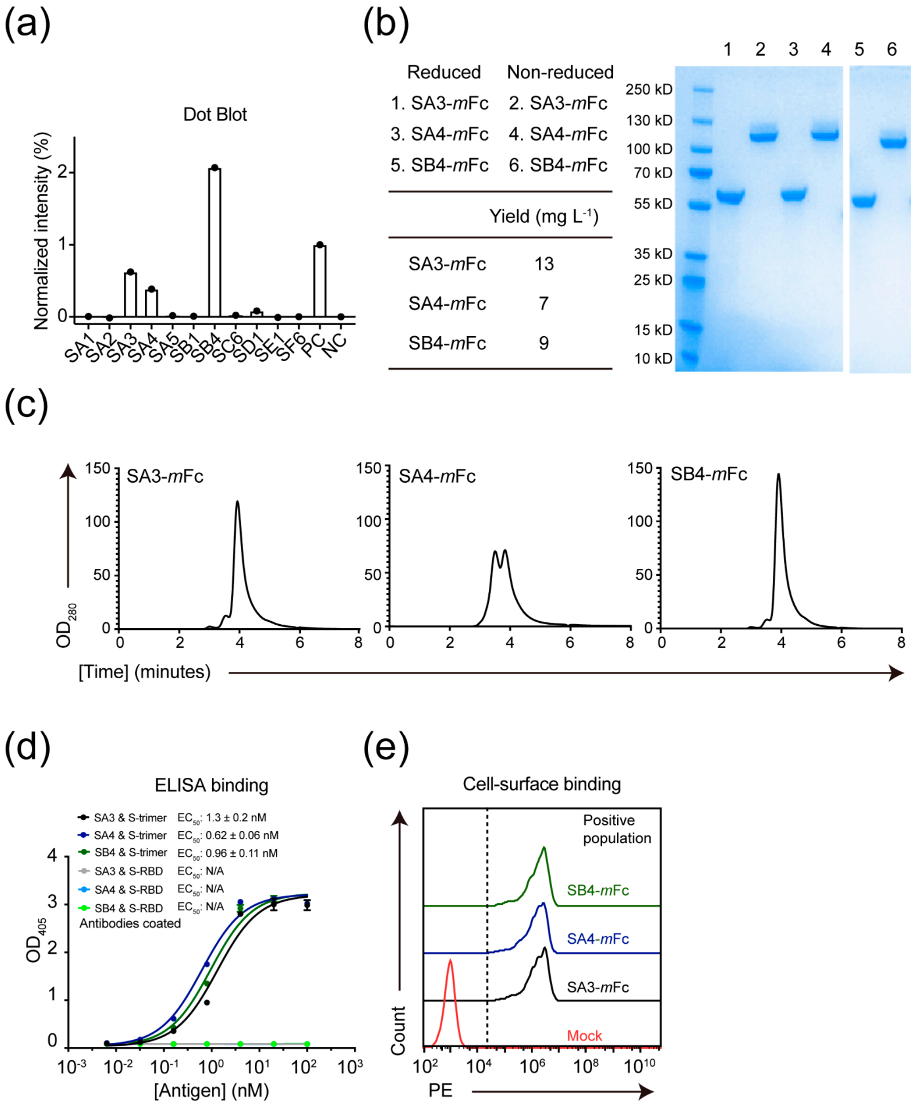

Figure S1. Candidate antibodies bind to spike of SARS-CoV-2 in non-RBD regions. **(a)** Dot Blot of these 11 unique antibodies expressing-supernatant were evaluated. Intensity was normalized by the signal of a purified *m*Fc-tag antibody used as a positive control. **(b)** Expression yield of purified SA3, SA4, and SB4 in scFv-*m*Fc format are shown. Three candidate antibodies in scFv-mouse Fc format were analyzed by SDS-PAGE, in non-reduced and reduced forms. **(c)** HPLC-SEC assessment of these three candidate antibodies stability. Aliquots of antibodies protein dissolved in PBS buffer (150 mM NaCl, 20 mM sodium phosphate, pH 7.4) were incubated at 4 ºC for 21 days and subjected to analysis in an Agilent Bio SEC-5, 500A HPLC system running with the same PBS buffer at a flow rate of 0.5 mL min^{-1}. **(d)** Binding ELISA for the three candidate antibodies against recombinant, biotinylated SARS-CoV-2 spike protein (S-trimer-His) and SARS-CoV-2 spike RBD protein (S-RBD-His) (n=3). EC_{50} of different antibodies are shown. **(e)** HEK293T cells transfected with expression plasmid encoding the full-length spike of SARS-CoV-2 were incubated with purified three antibodies and stained with PE labelled anti-human Fc secondary antibody, then analyzed by FACS.

Figure S2

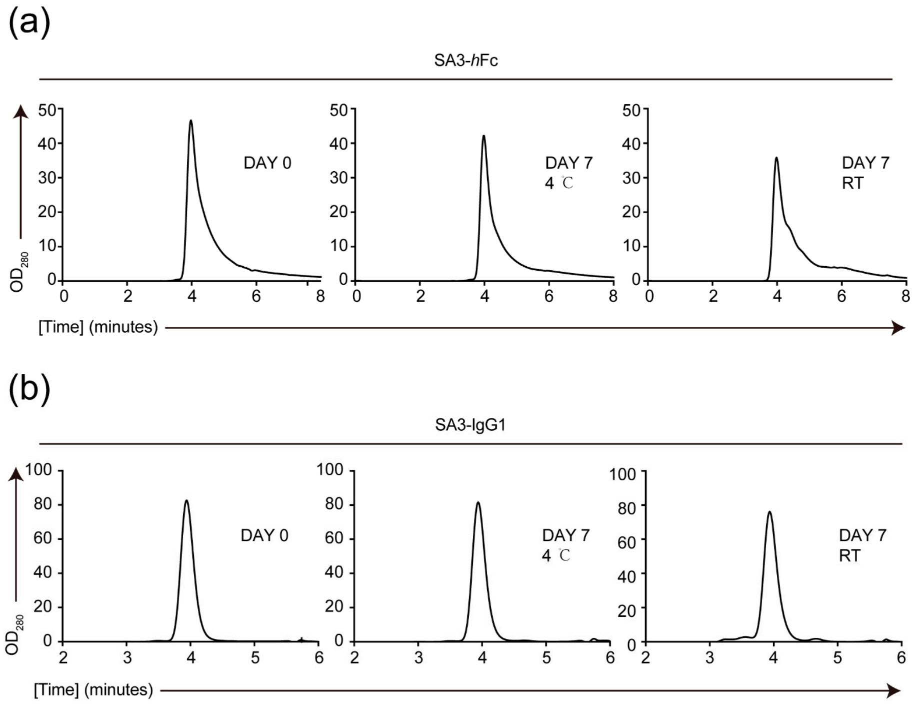

Figure S2. HPLC-SEC thermostability assay of SA3-*h*Fc and SA3-IgG1. HPLC-SEC assessment of **(a)** SA3-*h*Fc and **(b)** SA3-IgG1 thermal stability. Aliquots of SA3 protein dissolved in PBS buffer (150 mM NaCl, 20 mM sodium phosphate, pH 7.4) were incubated at 4 ºC or room temperature (RT) for 7 days and subjected to analysis in an Agilent Bio SEC-5, 500A HPLC system running with the same PBS buffer at a flow rate of 0.5 mL min^{-1}.

Figure S3

(a)

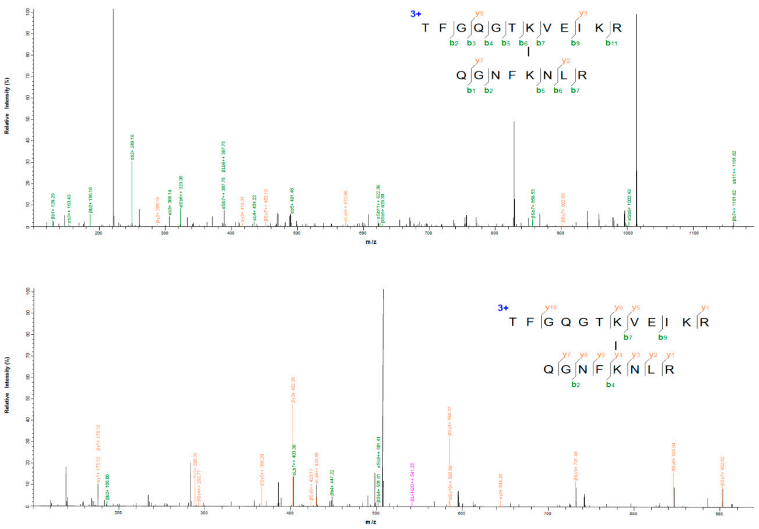

(b)

MS³ of TFGQGTKVEIKR

b				y
---	1	T	12	---
249.1234	2	F	11	1262.7215
306.1448	3	G	10	1115.6531
434.2034	4	Q	9	1058.6317
491.2249	5	G	8	930.5731
592.2726	6	T	7	873.5516
720.3675	7	K	6	772.5039
819.4359	8	V	5	644.409
985.67(+DSSO moiety-NH_3)	9	E	4	545.3406
1061.5626	10	I	3	416.298
1189.6575	11	K	2	303.2139
---	12	R	1	175.119

MS³ of QGNFKNLR

b				y
---	1	Q	8	---
186.0873	2	G	7	848.4737
283.15(-NH_3)	3	N	6	877.53(+DSSO moiety)
447.1987	4	F	5	763.48(+DSSO moiety)
575.2936	5	K	4	530.3409
689.3365	6	N	3	402.2459
802.4206	7	L	2	288.203
---	8	R	1	175.119

Figure S3. SA3 antibody cross-linked with SARS-CoV-2 spike via TFGQGTKVEIKR and QGNFKNLR peptides. **(a)** Tertiary MS for individual peptides TFGQGTKVEIKR and QGNFKNLR. **(b)** Peptide mass fingerprint for TFGQGTKVEIKR and QGNFKNLR.

Figure S4

(a)

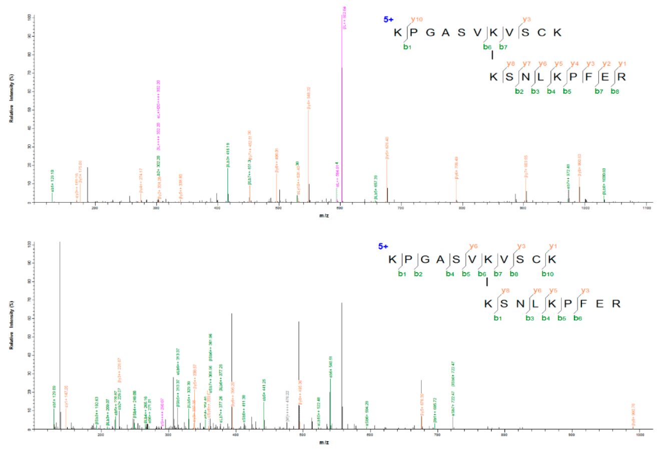

(b)

MS^3 of KPGASVKVSCK

b				y
---	1	K	11	---
226.155	2	P	10	1118.61(+Carbamidomethyl+DSSO moiety)
283.1765	3	G	9	1021.55(+Carbamidomethyl+DSSO moiety)
354.2136	4	A	8	821.455
441.2456	5	S	7	750.4178
540.314	6	V	6	663.3858
722.47(+DSSO moiety)	7	K	5	675.46(+Carbamidomethyl+DSSO moiety)
767.4774	8	V	4	493.35(Carbamidomethyl)
854.5094	9	S	3	394.15(+Carbamidomethyl)
957.5186	10	C	2	307.2(+Carbamidomethyl)
---	11	K	1	147.1128

MS^3 of KSNLKPFER

b				y
---	1	K	9	---
270.18(+DSSO moiety)	2	S	8	990.5367
330.1772(+DSSO moiety)	3	N	7	903.5047
529.38(+DSSO moiety)	4	L	6	789.4618
571.3562	5	K	5	676.3777
668.409	6	P	4	548.2827
815.4774	7	F	3	451.23
944.52	8	E	2	304.1615
---	9	R	1	175.119

Figure S4. SA3 antibody cross-linked with SARS-CoV-2 spike via KPGASVKVSCK and KSNLKPFER peptides. **(a)** Tertiary MS for individual peptides KPGASVKVSCK and KSNLKPFER. **(b)** Peptide mass fingerprint for KPGASVKVSCK and KSNLKPFER.

Figure S5

(a)

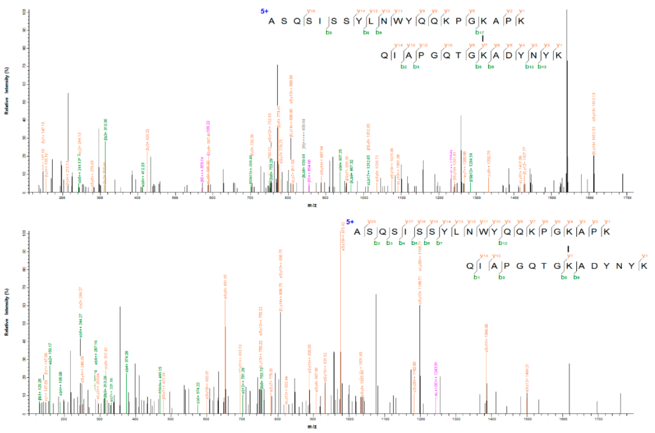

(b)

MS³ of ASQSISSYLNWYQQKPGKAPK

b				y
---	1	A	21	---
159.0764	2	S	20	2310.1877(+DSSO moiety)
287.135	3	Q	19	2223.1557
356.23(-H$_2$O)	4	S	18	2095.0971
487.2511	5	I	17	2008.0651(+DSSO moiety)
574.2831	6	S	16	1894.981(+DSSO moiety)
661.3151	7	S	15	1807.949
824.3785	8	Y	14	1720.917
937.4625	9	L	13	1557.8536
1051.5055	10	N	12	1482.0(+DSSO moiety-NH$_3$)
1237.5848	11	W	11	1330.7266
1400.6481	12	Y	10	1198.71(+DSSO moiety)
1528.7067	13	Q	9	981.584
1656.7653	14	Q	8	853.5254
1784.8602	15	K	7	725.4668
1881.913	16	P	6	597.3719(+DSSO moiety)
1938.9345	17	G	5	500.3191
2067.0294	18	K	4	443.2976
2138.0665	19	A	3	315.2027
2235.1193	20	P	2	244.1656
---	21	K	1	147.1128

MS³ of QIAPGQTGKIADYNYK

b				y
---	1	Q	16	---
242.1499	2	I	15	1638.8486
313.187	3	A	14	1612.13(+DSSO moiety)
410.2398	4	P	13	1540.88(+DSSO moiety)
467.2613	5	G	12	1443.79(+DSSO moiety)
595.3198	6	Q	11	1386.66(+DSSO moiety)
696.3675	7	T	10	1258.82(+DSSO moiety)
753.389	8	G	9	1071.5469(+DSSO moiety)
881.4839	9	K	8	1014.5255
994.568	10	I	7	886.4305
1065.6051	11	A	6	773.3464
1180.6321	12	D	5	702.3093
1343.6954	13	Y	4	587.2824
1543.51(+DSSO moiety)	14	N	3	424.2191
1689.19(+DSSO moiety-H$_2$O)	15	Y	2	310.1761
---	16	K	1	147.1128

Figure S5. SA3 antibody cross-linked with SARS-CoV-2 spike via ASQSISSYLNWYQQKPGKAPK and QIAPGQTGKIADYNYK peptides. **(a)** Tertiary MS for individual peptides ASQSISSYLNWYQQKPGKAPK and QIAPGQTGKIADYNYK. **(b)** Peptide mass fingerprint for ASQSISSYLNWYQQKPGKAPK and QIAPGQTGKIADYNYK.

Figure S6

(a)

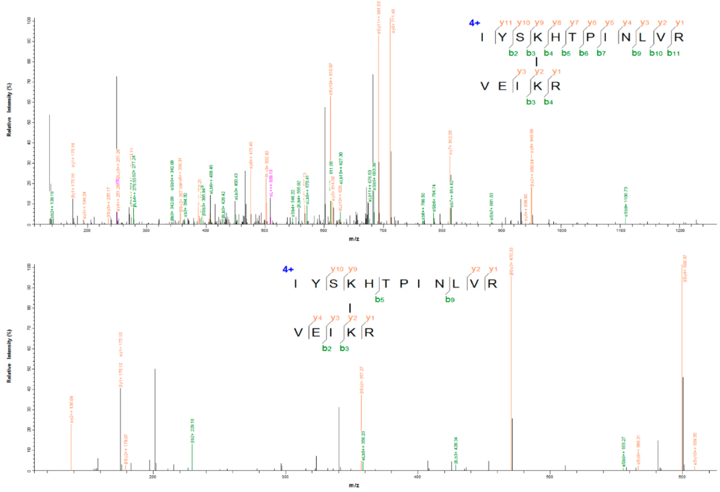

(b)

MS³ of IYSKHTPINLVR

b				y
---	1	I	12	---
277.1547	2	Y	11	682.55(charge2,+DSSO moiety-H_2O)
364.1867	3	S	10	1250.83(+DSSO moiety)
492.2817	4	K	9	1077.6527
629.3406	5	H	8	949.5578
730.3883	6	T	7	812.4989
827.441	7	P	6	711.4512
940.5251	8	I	5	614.3984
1054.568	9	N	4	501.3144
1167.6521	10	L	3	387.2714
1266.7205	11	V	2	274.1874
---	12	R	1	175.119

MS³ of VEIKR

b				y
---	1	V	5	---
229.1183	2	E	4	599.37(+DSSO moiety)
342.2023	3	I	3	470.33(+DSSO moiety)
470.2973	4	K	2	357.27(+DSSO moiety)
---	5	R	1	175.119

Figure S6. SA3 antibody cross-linked with SARS-CoV-2 spike via IYSKHTPINLVR and VEIKR peptides. **(a)** Tertiary MS for individual peptides IYSKHTPINLVR and VEIKR. **(b)** Peptide mass fingerprint for IYSKHTPINLVR and VEIKR.

Figure S7

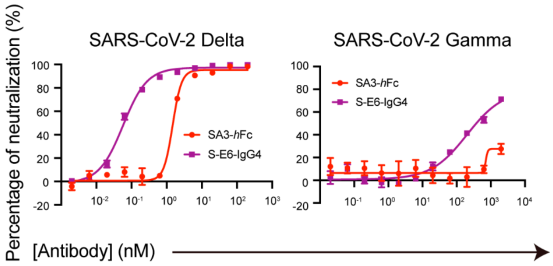

NT$_{50}$ (nM)	Delta	Gamma
SA3-*h*Fc	1.5 ± 0.2	> 500
S-E6-IgG4	0.058 ± 0.003	> 100

Figure S7. Neutralization of SA3-hFc against Delta and Gamma pseudovirus (PSV) strains. Dose-dependent neutralization of SA3-*h*Fc against SARS-CoV-2 Delta and Gamma PSVs were performed in triplicates (*n*=3). The apparent NT$_{50}$ values are determined by Hill analyses using one-way ANOVA of GraphPad Prism.

Figure S8

(a)

```
IGHV1-18    CDR-H1   CDR-H2              CDR-H3

SA3         DYGIS    WVSTYNVDTNYAQKFQG   --------DEVGSDWLGR-----Y  11
COV2-2034   TYGIS    WISAYNGNSNYGKKFQG   DLPIKVVVPAADYNWFDP------  18
CV42        SYGIS    WISAYNGNTNYAQKVQG   DRGYAATFGVFDY-----------  13
C1636       SYGIS    WISAHNGNTNYAQKFQG   -------FLALTYYYDSSGYPFDY  17
C1736       SYGIS    WISGYDGNTNYAQKVQG   DHRTVTTFGTFDY-----------  13
C1771       NYGIS    WSSVDNGHTNYAQKFQA   DHRTVVTFGTFDY-----------  13
R616-1D2    NYGLS    WISGNNGNTNYAQKFQG   DQSTVVTFGTFDY-----------  13
             **:*    * *  :  .:**.:*.*.
```

(b)

```
IGKV1-39    CDR-L1        CDR-L2     CDR-L3

SA3         RASQSISSYLN   AASSLQS    QQSYSTPRT--   9
COV2-2034   RASQSISSYLN   AASSLQS    QQSYSTPPT--   9
CV42        RASQSISSYLN   AASSLQS    QQTYITAFT--   9
C1636       RASQSISSYLN   AASSLQS    QQSYSTPRMYT   11
C1736       RASQSIGYYLN   AAASLQS    QQSYSTPFT--   9
C1771       RASHNINTFLN   APSILQS    QQTYRTPFT--   9
R616-1D2    RASQNINRYLN   AASGVQS    QQSYITPFT--   9
            ***:.*. :**   *  :  :**  **:*  *
```

Figure S8. Amino acid sequence analysis of SA3 antibody. **(a)** Multiple sequence alignment of heavy chain between SA3 and IGHV1-18/IGKV1-39 antibodies against the non-RBD spike of SARS-CoV-2. Antibody sequences were obtained from the CoV-AbDab database of the University of Oxford and aligned using CLUSTAL O (1.2.4) multiple sequence alignment. **(b)** Multiple sequence alignment of light chain between SA3 and IGHV1-18/IGKV1-39 antibodies against the non-RBD spike of SARS-CoV-2. Antibody sequences were obtained from the CoV-AbDab database of the University of Oxford and aligned using CLUSTAL O (1.2.4) multiple sequence alignment. Antibody complementarity determining regions (CDRs) were analyzed by abYsis.

Table S1

Table S1. The germline information and somatic hypermutations numbers (mutations) of heavy chain or light chain of the selected clones.

Clone	HC Germline	HC Mutations	LC Chain	LC Germline	LC Mutations
SA1	5-51	13	Kappa	3-20	5
SA2	3-30	7	Lambda	6-57	8
SA3	1-18	11	Kappa	1-39	0
SA4	1-18	11	Kappa	1-39	4
SA5	1-69	12	Lambda	5-45	5
SB1	3-30	7	Lambda	6-57	8
SB4	1-18	11	Kappa	1-39	6
SC6	1-18	11	Kappa	3-20	2
SD1	3-30	6	Lambda	5-45	6
SE1	3-30	11	Lambda	1-47	14
SF6	3-30	7	Lambda	6-57	8

Antibody sequences were analyzed by IMGT/V-QUEST database.
